# High-Throughput Biochemical Fingerprinting of Oleaginous *Aurantiochytrium* sp. Strains by Fourier Transform Infrared Spectroscopy (FT-IR) for Lipid and Carbohydrate Productions

**DOI:** 10.3390/molecules24081593

**Published:** 2019-04-22

**Authors:** Xin-Jun Yu, Chang-Yi Huang, Hong Chen, Dong-Sheng Wang, Jing-Liang Chen, Hui-Juan Li, Xiao-Yan Liu, Zhao Wang, Jie Sun, Zhi-Peng Wang

**Affiliations:** 1 Key Laboratory of Bioorganic Synthesis of Zhejiang Province, College of Biotechnology and Bioengineering, Zhejiang University of Technology, No.18, Chaowang Road, Hangzhou 310014, Zhejiang, China; xjyu@zjut.edu.cn (X.-J.Y.); hcy929390419@gmail.com (C.-Y.H.); hzchenhong1016@163.com (H.C.); jlchen2018@163.com (J.-L.C.); hzwangzhao@sina.cn (Z.W.); jsun@zjut.edu.cn (J.S.); 2 Institute of Biological Resources, Jiangxi Academy of Sciences, Nanchang 330096, Jiangxi, China; 3 Department of Bioengineering, College of Chemical and Environmental Engineering, Shandong University of Science and Technology, Qingdao 266590, China; lihuijuan611@126.com; 4 Jiangsu Key Laboratory for Biomass-based Energy and Enzyme Technology, Huaiyin Normal University, Huaian 223300, Jiangsu, China; catty5082003@163.com; 5 Key Laboratory of Sustainable Development of Polar Fishery, Ministry of Agriculture and Rural Affairs, Yellow Sea Fisheries Research Institute, Chinese Academy of Fishery Sciences, Qingdao 266071, Shandong, China; wangzp@ysfri.ac.cn

**Keywords:** *Aurantiochytrium* sp., carbohydrate, DHA, feed, FT-IR, high-throughput

## Abstract

The traditional biochemical methods for analyzing cellular composition of oleaginous microorganisms are time-consuming, polluting, and expensive. In the present study, an FT-IR method was used to analyze the cellular composition of the marine oleaginous protist *Aurantiochytrium* sp. during various research processes, such as strains screening, medium optimization, and fermentation, and was evaluated as a green, low-cost, high throughput, and accurate method compared with the traditional methods. A total of 109 *Aurantiochytrium* sp. strains were screened for lipid and carbohydrate production and the best results were found for the strains No. 6 and No. 32. The yields and productivities could reach up to 47.2 g/L and 0.72 g/L/h for lipid, 21.6 g/L and 0.33 g/L/h for docosahexaenoic acid (DHA) in the strain No. 6, and 15.4 g/L and 0.18 g/L/h for carbohydrate in the strain No. 32, under the optimal conditions, respectively. These results confirmed potentials of the two *Aurantiochytrium* sp. strains for lipid, DHA, and carbohydrate productions at industrial scales. The FT-IR method in this study will facilitate research on the oleaginous *Aurantiochytrium* sp., and the obtained two strains for lipid and carbohydrate productions will provide the foundations for their applications in medical, food, and feed industries.

## 1. Introduction

Docosahexaenoic acid (DHA), a kind of polyunsaturated fatty acids (PUFAs), plays essential roles in alleviating cardiovascular diseases, hypertension, diabetes, neuropsychiatric disorders, and cancer in humans [1]. Moreover, aquatic animals and poultries also require DHA for their development and survival [2]. *Aurantiochytrium* sp. is a kind of *Thraustochytrids*, which belong to a group of heterotrophic, unicellular, marine protists, and *Aurantiochytrium*, *Oblongichytrium*, and *Schizochytrium* in *Thraustochytrids* are considered to form one genus [3]. As a well-known oleaginous microorganism, *Aurantiochytrium* sp. can accumulate a large amount of lipid, rich in DHA in its cell. Thus, *Aurantiochytrium* sp. is a DHA producer and a substitute for the traditional fish oil resource as a new DHA resource [4]. Moreover, cells of *Aurantiochytrium* sp. are rich in valuable metabolites, such as terpenoids, pigment, carbohydrate, and protein, which have high nutritional values and multiple physiological functions. Thus, *Aurantiochytrium* sp. has been widely applied in the medical, food, and feed industries [5,6].

A large number of biochemical methods are essential for the analysis of the cellular compositions of *Aurantiochytrium* sp. in numerous types of researches, such as strain screening, medium optimization and fermentation monitoring for productions of the high-value metabolites. For examples, lipid content in the cell was analyzed through organic solvent extraction, evaporation and weighing [7,8]. Some cellular dyes, such as Sudan Black B and Nile red, were also used to dye lipid in the cell, and the fluorescence intensity was analyzed for lipid quantification [9,10]. A wet extraction method was directly exerted on the wet cells of *Aurantiochytrium* sp. for lipid extraction and analysis through a high shear mixer (HSM) [11]. Otherwise, pyrolysis of cell based on the thermogravimetric analysis and ultrasound-assisted transesterification in wet *Aurantiochytrium* sp. cells using potassium carbonate were also used to treat cell for analysis of bio-macromolecules [12,13]. The phenol-sulfuric acid method, DNS method, folin phenol method, sulfuric acid-anthrone method, Bradford method, etc., which relies on biomass extraction and needs a large amount of the biomass sample, were used for protein or carbohydrate quantification [14,15,16].

Moreover, large numbers of procedures, such as cell lyophilization and disruption, treatments using organic or inorganic reagents, the calibration standard, etc., were all needed in these traditional biochemical methods. In summary, these traditional biochemical methods described above are time-consuming, heavily polluting and require a large amount of equipment. Thus, there is a pressing need for developing reliable, environment-friendly and fast methods to analyze cellular composition in numerous researches for *Aurantiochytrium* sp.

FT-IR (Fourier Transform Infrared Spectroscopy) is molecular vibrational spectroscopy that dissects chemical functional groups in the mid-infrared zone between 4000 and 400 cm^−1^. The typical functional chemical groups in lipid, protein, and carbohydrate, such as C–H stretching vibration acyl chains, ester C=O stretching band, C=O stretching vibration, N–H bending vibration, C–O and C–O–C stretching band, and so on, have characteristic absorbance in different frequency regions of the mid-infrared zone. Moreover, FT-IR can directly scan the cultured microalgal cells for cellular composition analysis without complex procedures and organic or inorganic reagents which are necessities in the traditional biochemical methods [17]. Thus, FT-IR has been a rapid, green, high throughput and cost-effective tool for monitoring and analysis of the microalgal cellular composition [17,18]. The lipid content of *Thraustochytrium* sp., belonging to the *Thraustochytrids* same as *Aurantiochytrium* sp., has been analyzed by the FT-IR technology for comparing with the marine oleaginous yeast [19]. The *Schizochytrium* sp. cell was pyrolyzed, and the cellular lysates were analyzed by FT-IR method for cellular composition characterization [20]. The cellular composition of the *Aurantiochytrium* sp. KRS101, including protein, lipid, carbohydrate, and ash, was been analyzed through FT-IR method [21]. However, the feasibility and reliability of FT-IR method for analyzing cellular composition in *Aurantiochytrium* sp. have rarely evaluated comparing with the traditional biochemical methods. 

In the present study, an analytic method based on the FT-IR technology for the three main bio-macromolecules, including lipid, carbohydrate, and protein, will be constructed to analyze the cellular composition of the oleaginous protist *Aurantiochytrium* sp. This method will be applied in strain screening, medium optimization and fermentation of *Aurantiochytrium* sp. The feasibility and reliability of the FT-IR method and the potentials of *Aurantiochytrium* sp. strains for lipid and carbohydrate productions will be evaluated, which accelerates the research development of *Aurantiochytrium* sp., and improves its applications in medical, food and feed industries. 

## 2. Results and Discussion

### 2.1. FT-IR in Aurantiochytrium sp. Strains Screening

Screening *Aurantiochytrium* sp. strains with high-level content of lipid or carbohydrate is the first and most essential step for achieving their significant industrial value. However, traditional biochemical analysis of lipid and carbohydrate need a long time, a large amount of cell culture and multi-instruments, leading to time and space limitations for screening. FT-IR is a high-throughput method for cellular biomolecular analysis, and needs a trace amount of cell culture, enabling it to be used for the rapid and high- throughput screening of *Aurantiochytrium* sp. strains.

**Figure 1:**
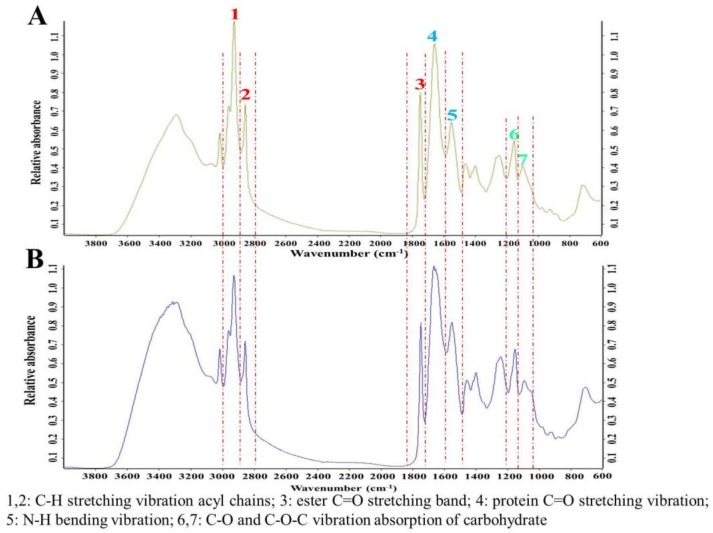
The profile of FT-IR absorption peaks for the H_2_O-treated *Aurantiochytrium* sp. cell (**A**) and the D_2_O-treated *Aurantiochytrium* sp. cell (**B**).

**Figure 2:**
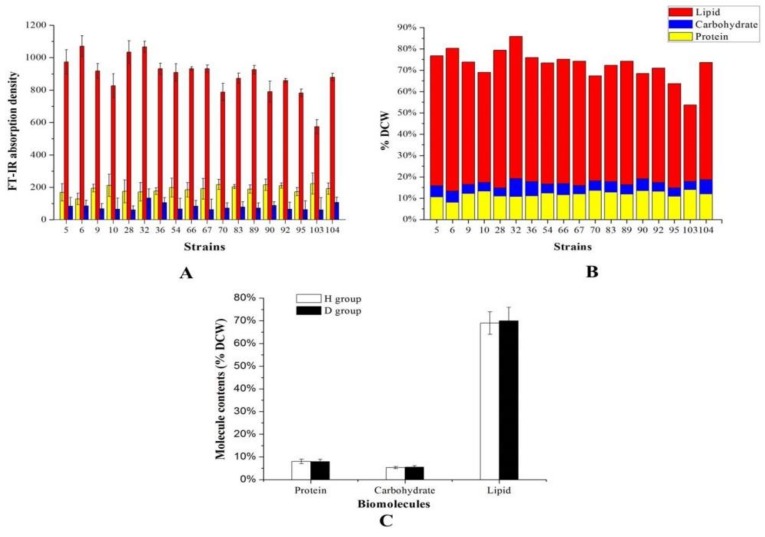
Three main bio-macromolecules contents of 18 *Aurantiochytrium* sp. strains deduced from the FT-IR analysis (**A**) and the traditional biochemical methods (**B**), and bio-macromolecules contents of cell between the H_2_O-treated group (H group) and the D_2_O-treated group (D group) for *Aurantiochytrium* sp. strain No. 6 (**C**). Data are given as means ± standard deviation, *n* = 3.

**Figure 3:**
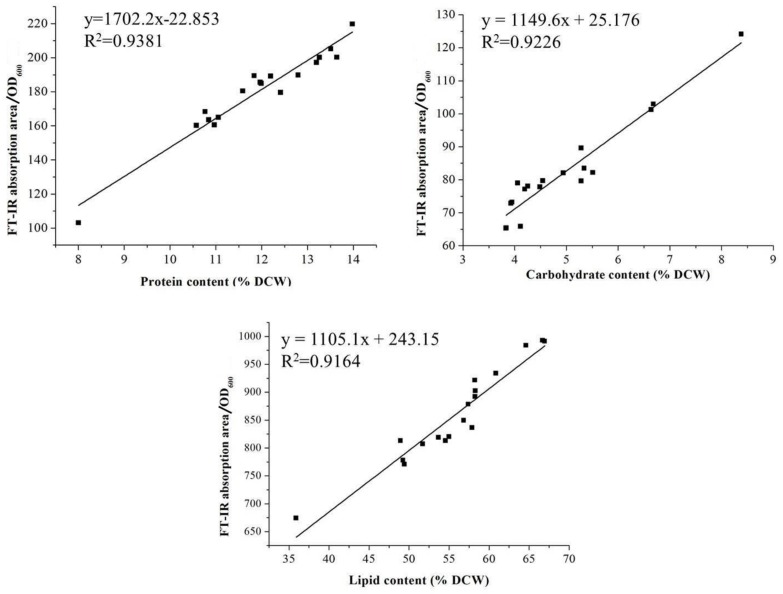
Linear correlations between the results deduced by the traditional and the FT-IR methods for the contents of the main three bio-macromolecules in *Aurantiochytrium* sp. strains.

Protein, carbohydrate, and lipid are three major bio-macromolecules of the cell and play different roles in the cell. Protein is closely related to basic biosynthesis and cell division, while lipids and carbohydrate serve as intracellular reservoirs of carbon and energy. Cells preferentially synthesize carbohydrate or lipid rather than protein for reserving carbon and energy to answer the poor growth condition. To screen *Aurantiochytrium* sp. strains synthesizing high level of carbohydrate and lipid, the medium with nitrogen limitation condition, which might increase the carbohydrate and lipid synthesis and lead to a reduced synthesis of the protein [22], was used in this study. A total of 109 strains in our *Aurantiochytrium* sp. strain library were analyzed by FT-IR. Based on the cellular contents of lipid, carbohydrate, and protein, a total of 18 strains were picked up among the 109 strains for further analysis after the 1st screening round (Data not shown). The FT-IR absorption profile for the *Aurantiochytrium* sp. strain was shown in Figure 1A. Lipid was the bio-macromolecule with the highest content in *Aurantiochytrium* sp. cell, followed by protein and carbohydrate in proper order, consistent with its oleaginous characteristic and similar to other *Aurantiochytrium* sp. strain [22]. Absorption areas of representing peaks were used to quantify the contents of protein, carbohydrate, and lipid. As shown in Figure 2A,B, the highest content of lipid was obtained in *Aurantiochytrium* sp. strain 6, while the lowest content of protein was obtained in this strain. The strain No. 32, with the highest content of carbohydrate, is a potential carbohydrate producer. Oppositely, the *Aurantiochytrium* sp. strain 103 had the highest content of protein in its cell, with the lowest content of lipid and carbohydrate. These results indicated that the contents of carbohydrate and lipid, and the content of protein were negatively correlated in *Aurantiochytrium* sp. cell. The traditional biochemical methods for analyzing lipid, carbohydrate, and protein in the *Aurantiochytrium* sp. cells were applied to validate the accuracy of FT-IR analysis (Figure 2B). To correct the effect of deuterated metabolic products on contents of three biomolecules in the *Aurantiochytrium* sp. cells, the *Aurantiochytrium* sp. strain No. 6 was cultured in the medium prepared with D_2_O for cell deuteration (D group). Meanwhile, the cells cultured in the medium prepared with H_2_O were set as the H group. As shown in Figure 1, the FT-IR profiles of the D- and H-group cells were very similar. Further quantification analysis deduced by the FT-IR showed that the contents of three biomolecules, including lipid, protein and carbohydrate, were similar for the D- and H-group cells, suggesting that deuteration of cell or deuterated metabolic products distributed in a cell cannot affect the FT-IR results (Figure 2C). Biomass composition of *Aurantiochytrium* sp. varied greatly among the 18 strains (Figure 3). The lipid made up 35.9–67.0% of DCW, the protein made up 8.0–14.0% of DCW, and the carbohydrate made up 3.8–8.4% of DCW. The FT-IR absorption area and the corresponding protein, carbohydrate and lipid content, were analyzed respectively. As shown in Figure 3, good linear correlations among FT-IR absorption area versus protein (R^2^ = 0.9381), carbohydrate (R^2^ = 0.9226) and lipid (R^2^ = 0.9164) contents, respectively, were observed in 18 *Aurantiochytrium* sp. strains. In this study, two normalization strategies, with amide I (1690–1650 cm^−1^, associated with biomass) and C–H stretching region (3000–2800 cm^−1^, associated with lipid) as internal reference peaks for assessments of the relative contents of protein, lipid and carbohydrate, respectively, were applied to minimize the peak absorption’s fluctuation caused by the inhomogeneous thickness of sample tablet [18,23]. However, weak linear correlations were obtained through these two normalization methods, indicating that the two normalization methods are highly species-specific among microorganism strains, and not suitable for *Aurantiochytrium* sp. strains (data not shown). As the Lambert-Beer’s law described, the lipid or carbohydrate content (% DCW) is proportional to the corresponding FT-IR areas normalized by the amide I band (A_lipid or carbohydrate_/A_amide I_) only when the protein content keeps unchanged if the ratio of the molar absorptive coefficients of protein and lipid or carbohydrate is a constant [24]. This law is not suitable in *Aurantiochytrium* sp. strain due to the high variations of protein content in its cellular composition (Figure 2). In the present study, we normalized the OD_600_ of every cell sample to 0.1 and then analyzed its FT-IR profile. There were good linear correlations between lipid, carbohydrate and protein content (%DCW) and the corresponding FT-IR areas normalized by the OD_600_ (A_lipid, carbohydrate or protein_/0.1 OD_600_), indicating that FT-IR analysis normalized by the OD_600_ can be used to analyze cellular composition of *Aurantiochytrium* sp. strains. The same method was also applied to normalize the FT-IR peaks to analyze the lipid, carbohydrate and protein content in algae, yeast, and bacteria cell [25].

### 2.2. Optimizations of Media for Lipid and Carbohydrate Productions

**Figure 4:**
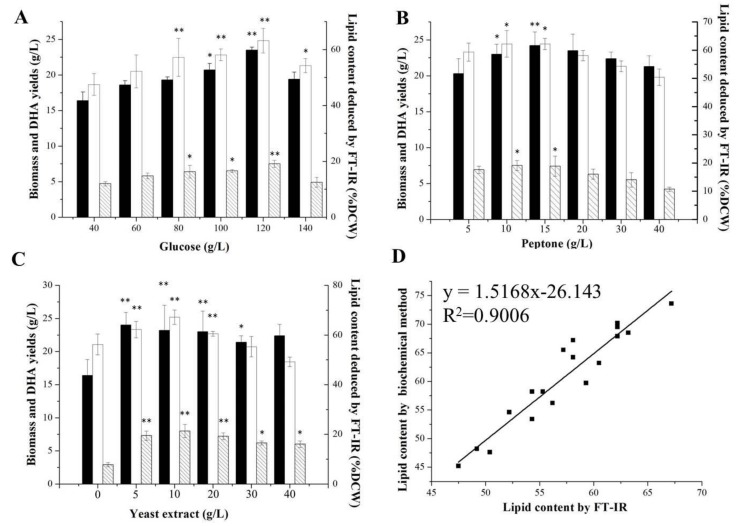
Different concentrations of glucose, peptone and yeast extract on biomass, lipid and DHA productions in *Aurantiochytrium* sp. strain No. 6 and evaluation of the related FT-IR results. (**A**), Effects of concentrations of glucose on biomass, lipid and DHA synthesis; (**B**), Effects of concentrations of peptone on biomass, lipid and DHA synthesis; (**C**), Effects of concentrations of yeast extract on biomass, lipid and DHA synthesis; (**D**), the linear relationship between the lipid contents deduced by the traditional method and the FT-IR method. Data are given as means ± standard deviation, *n* = 3. Filled blank, biomass; White blank, lipid content; blank with diagonal, DHA yield. * *p* < 0.05; ** *p* < 0.01 The yield under the medium composed of 40 g/L glucose, 10 g/L peptone, and 5 g/L yeast extract was set as the control.

*Aurantiochytrium* sp. strain 6 had been screened for lipid and DHA productions; however, the media composition needed to be further optimized to obtain the optimal yields. As shown in Figure 4, the optimal lipid content and DHA yield of *Aurantiochytrium* sp. strain No. 6 were 73.6% DCW and 8 g/L when the concentrations of glucose, peptone and yeast extract were 120, 15 and 10 g/L, 35.1% and 25% higher than those in the initial condition. *Aurantiochytrium* sp. strain 6 could endure up to 120 g/L glucose, and did not exhibit the glucose inhibition until the concentration of glucose reached up to 140 g/L, which was higher than those of the other *Aurantiochytrium* sp. strains, and characterized the *Aurantiochytrium* sp. strain 6 as an ideal lipid and DHA producer needing less fed-batch times with lower potential pollution risk [26,27]. C/N ratio in the medium is a key factor regulating lipid synthesis in oleaginous microorganisms, and the high C/N ratio condition (Nitrogen limitation) induces improvement of lipid production for carbon and energy reservations as an anti-stress response. Under nitrogen limitation condition, residual carbon source in medium flow to the lipid synthesis by producing acetoacetyl coenzyme A, which was the structural unit for lipid and DHA synthesis. However, a high C/N ratio is a disadvantage to cell growth and protein synthesis [28]. The optimal C/N ratio of the cultivation medium for lipid and DHA synthesis in strain 6 was up to 4.8 (glucose as carbon resource, peptone and yeast extract as nitrogen resource), which induced lipid and DHA synthesis significantly and had no obvious negative effect on cell growth (Figure 4). Huang et al. found that a low C/N ratio (1.25) was optimal for *Aurantiochytrium limacinum* SR21 to produce lipid and DHA at a balance condition between biomass and lipid synthesis [28]. Results reported by Ryu et al. indicated that the highest DHA productivity (38.8% of total lipid) was observed in *Aurantiochytrium* sp. KRS101 when a C/N ratio in the medium was up to 20 [29]. These results validated that the C/N ratio in medium plays a significant role in regulating biomass, lipid and DHA productions in *Aurantiochytrium* sp., and showed strain-specific among different strains. 

**Figure 5:**
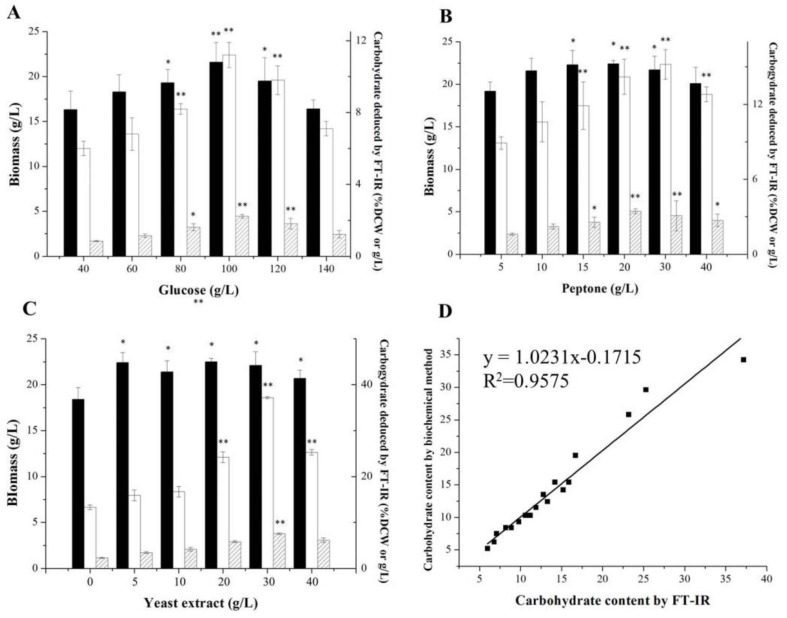
Different concentrations of glucose, peptone and yeast extract on biomass and carbohydrate productions in *Aurantiochytrium* sp. strain No. 32 and evaluation of the related FT-IR results. (**A**), Effects of concentrations of glucose on biomass and carbohydrate production; (**B**), Effects of concentrations of peptone on biomass and carbohydrate production; (**C**), Effects of concentrations of yeast extract on biomass and carbohydrate production; (**D**), the linear relationship between the carbohydrate contents deduced by the traditional method and the FT-IR method. Data are given as means ± standard deviation, *n* = 3. Filled blank, biomass; White blank, carbohydrate content; blank with diagonal, carbohydrate yield. * *p* < 0.05; ** *p* < 0.01 The yield under the medium composed of 40 g/L glucose, 10 g/L peptone, and 5 g/L yeast extract was set as the control.

*Aurantiochytrium* sp. strain No. 32 had been screened for the high carbohydrate content in its cells, and the medium composition was further optimized for carbohydrate production. As shown in Figure 5, the highest carbohydrate content and yield (34.2% DCW, 7.56 g/L) were obtained under the optimal medium with 100 g/L glucose, 20 g/L peptone and 30 g/L yeast extract, 307% and 367% higher than those in the initial condition. As a heterotrophic marine protist, *Aurantiochytrium* sp. strain No. 32 could utilize up to 100 g/L of glucose to grow fast (22.1 g/L of biomass in 4 days) without glucose effect and inhibition, making it an ideal strain for industrial fermentation purposes. Meanwhile, the carbohydrate content and yield in *Aurantiochytrium* sp. strain No. 32 cell could reach up to 34.2% of DCW and 7.56 g/L, which were higher than those in *A. limacinum* SR21 cell (33.38% DCW, 3.84 g/L) and those in *Schizochytrium* sp. HX-308 (13.58% DCW, 6.63 g/L) [22,30]. Under nutrient limitations, microbial cell preferentially synthesized carbohydrate and lipid for the carbon and energy reservation. However, accumulation of lipid was enhanced more quickly than that of carbohydrate in *Aurantiochytrium* sp. under different nutrient limitation conditions [22]. Thus, a relatively low C/N ratio of 2 was the optimal value for balancing carbohydrate synthesis and biomass in *Aurantiochytrium* sp. strain No. 32 to avoid over-accumulation of lipid, lower than that (4.8) of *Aurantiochytrium* sp. strain 6 for lipid production. Meanwhile, many micro-elements, such as metal elements and vitamins, were essential for *Aurantiochytrium* sp. strains for cell growth and metabolite synthesis, increasing the production cost significantly [31]. Unlike the other strains, *Aurantiochytrium* sp. strain No. 32 did not need any micro-elements to obtain a high level of biomass and carbohydrate in its cell. This result indicates that the *Aurantiochytrium* sp. strain No. 32 is an ideal potential candidate for producing carbohydrate. 

Moreover, the FT-IR method was also used for lipid and carbohydrate contents analysis in optimization performances, and the results (% DCW) from the FT-IR method and the traditional biochemical method had high linear correlations with R^2^ values of 0.9006 for lipid analysis and 0.9575 for carbohydrate analysis, respectively (Figure 4 and Figure 5). The linear equations representing lipid and carbohydrate contents with the FT-IR method (x-axis) vs the traditional biochemical method (y-axis) were y = 1.5168x − 26.143 for lipid and y = 1.0231x − 0.1715 for carbohydrate (Figure 4 and Figure 5). The area of the 1740 cm^−1^ in FT-IR, representing the ester C=O stretching band, was used to quantify the lipid content in *Thraustochytrium* sp. cells [19]. Meanwhile, the 3000–2800 cm^−1^ area of FT-IR, representing the C–H stretching vibration acyl chains, was also used to analyze the lipid contents in marine microalgae with the amino I area (1650 cm^−1^) as the internal standard. However, this range of area in FT-IR could not be used to quantify the lipid content in some microalgae due to its high variability in this area of FT-IR caused by the overlapping of OH bond absorption and therefore leading to misinterpretations [32]. However, these two areas in FT-IR, representing two important chemical bonds in lipid, were firstly considered in combination for lipid analysis in *Aurantiochytrium* sp. cells due to its high lipid content and relative stable profile in the 3000–2800 cm^−1^ of FT-IR, and a high accuracy for lipid analysis (R^2^ = 0.9006) was obtained through this analysis method. However, these results above were obtained at an approximate lipid content range of 45–73% DCW, cells with the lipid content under 26% DCW might not be analyzed by this FT-IR method. This result indicated that the FT-IR method for lipid analysis used in this study is specific for the oleaginous microorganism, such as *Aurantiochytrium* sp. (with higher lipid content, up to 70% in cells), while not fit for the microorganisms with low lipid content. 

For carbohydrate analysis, absorption signals around 1180–1133 cm^−1^ in FT-IR characterized over a C–O, and the C–O–C stretching band area was used to quantify the total carbohydrate content in the cell. The C–O and C–O–C stretching bands were the typical bonds in carbohydrate, and a high coefficient of determination (0.9575) was obtained through this method, indicating that the 1200–900 cm^−1^ in FT-IR response accurately to the carbohydrate content in the *Aurantiochytrium* sp. cells. Absorption area of around the 1200–900 cm^−1^ band in FT-IR was also used to analyze carbohydrate content in *Chlorella pyrenoedosa*, *Nannochloropsis* sp., *Botryococcus braunii* and *Microcystis aeruginosa* [33]. 

### 2.3. Assessments of the FT-IR and Traditional Methods

Assessments of the FT-IR and traditional methods for analysis of bio-macromolecule were summarized in Table 1. At least 1 g of the dried cell is needed for analysis using the traditional biochemical method; thus, at least 50 mL of liquid sample was cultured and lyophilized. The time consumption of this method for analysis is at least 72 h, including cell lyophilization and grinding, lipid extraction and weighing, carbohydrate extraction, reaction with sulfuric acid and anthrone, and analysis of carbohydrate content. A large number of reagents, such as liquid nitrogen, chloroform, methanol, sulfuric acid, and anthrone, which have severe corrosivity, irritation, and pollution, have to be used in the traditional biochemical method. Moreover, numerous pieces of equipment, such as the centrifuge, freeze dryer, mortar, pestle, evaporimeter, and balance, are also essential for the traditional method. Thus, the traditional biochemical method for bio-macromolecules is time-consuming, expensive, equipment-limited and severely polluted. The 96 well microplates are used for analysis in FT-IR. Therefore, only approximately 200 μL of the liquid sample is needed for every sample analysis through FT-IR. Without cell lyophilization, bio-macromolecule extraction and analysis, only approximate 4 h is required for FT-IR analysis, saving approximate 17 times than that of the traditional biochemical method. Moreover, due to the simple process, only the FT-IR spectrometer and spectrophotometer are needed in FT-IR, and no chemical reagent is required for analysis. Unlike the traditional biochemical methods, which can detect only one kind of bio-macromolecule at a time, the FT-IR can simultaneously detect the content of lipid, carbohydrate and protein components. However, the overlap of the stretching vibration peaks of different biochemical components made FT-IR analysis a certain degree of quantity inaccuracy. In this study, various combinations of peaks-integration and normalization were analyzed and compared with the results from the traditional biochemical methods, and high coefficients of determination (R = 0.9006 for lipid and R = 0.9575 for carbohydrate) were obtained. Thus, FT-IR analysis is a very economical and green method with less pollution and high accuracy for bio-macromolecule analysis in *Aurantiochytrium* sp. cell.

### 2.4. FT-IR for Monitoring Fermentative Parameters during Lipid and Carbohydrate Productions

**Figure 6:**
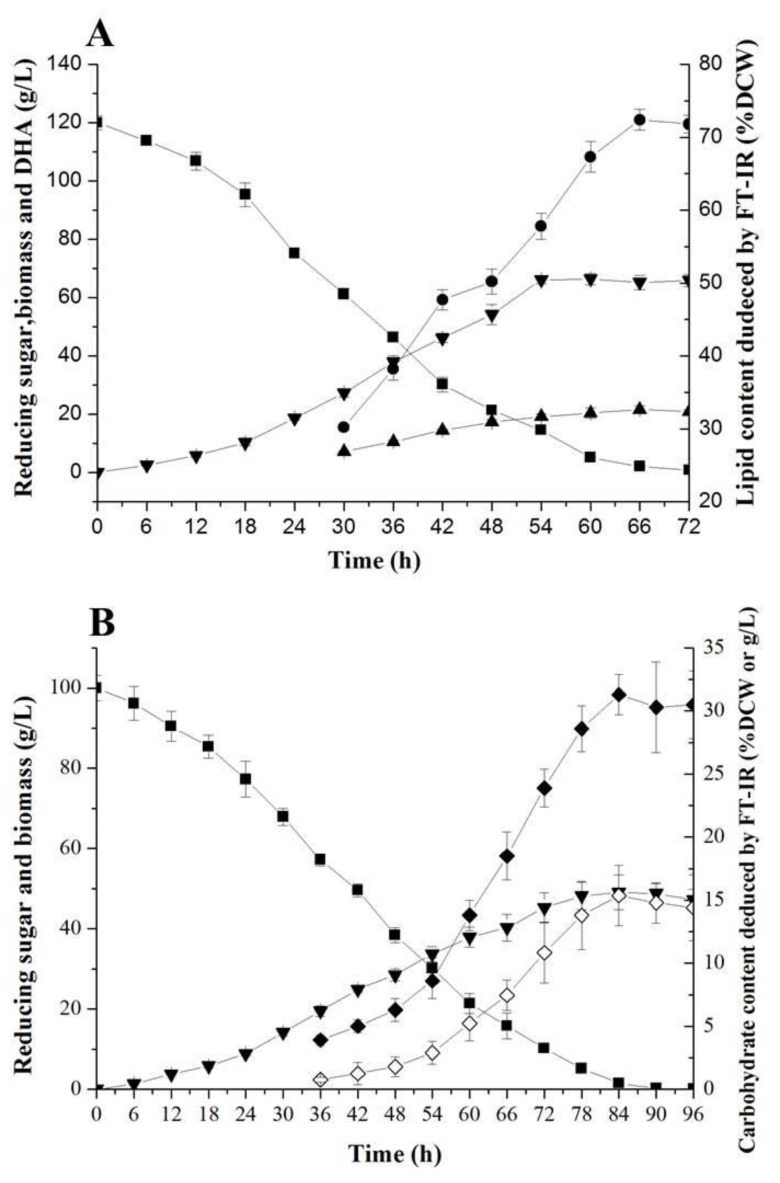
The fermentative performances of the *Aurantiochytrium* sp. strains No. 6 and No. 32 for lipid and carbohydrate productions under FT-IR monitoring. (**A**), lipid and DHA production by *Aurantiochytrium* sp. strains No. 6; (**B**), carbohydrate production by *Aurantiochytrium* sp. strains No. 32. Reducing sugar, filled squares; biomass, filled inverted triangles; lipid content, filled circles; lipid yield, filled triangles; carbohydrate content, filled diamonds; carbohydrate yield, open diamonds.

Based on the optimized media for the *Aurantiochytrium* sp. strains No. 6 and No. 32, respectively, 5 L fermentations were performed for lipid and carbohydrate productions under the FT-IR monitoring. As shown in Figure 6A, a total of 119.1 g/L reducing sugar was consumed, and a maximum DCW of up to 66.4 g/L was obtained in 60 h. The cell of *Aurantiochytrium* sp. strains No. 6 entered the stable stage of growth at 54 h and was kept until the end of fermentation at 72 h. At the later period of fermentation, the maximum lipid content and DHA yield, up to 72.4% DCW and 21.6 g/L, respectively, were obtained at 66 h, suggesting that the carbon resource was first consumed for cell growth of *Aurantiochytrium* sp. strains No. 6, and then for lipid synthesis when the carbon resource was consumed to deficiency. The DCW, lipid and DHA productivities of *Aurantiochytrium* sp. strain No. 6 could reach up to 1.11 g/L/h, 0.72 g/L/h and 0.33 g/L/h, respectively, higher than those of *Aurantiochytrium* sp. strains YLH70, SR21, and *Japonochytrium marinum* AN-4 [26,34]. As an essential fermentative parameter of oleaginous *Aurantiochytrium* sp., lipid content was monitored through FT-IR method synchronously, guiding for lipid and DHA production of *Aurantiochytrium* sp. with high speed and accuracy. 

FT-IR method was also used for carbohydrate analysis during the fermentation by *Aurantiochytrium* sp. strain No. 32. As shown in Figure 6B, a total of 99.9 g/L of reducing sugar was consumed during the fermentation process (96 h), with a consumption rate of up to 1.04 g/L/h, indicating an ideal fermentative characteristic of *Aurantiochytrium* sp. strain No. 32 without apparent glucose inhibition effect. The cell of *Aurantiochytrium* sp. strain No. 32 reached the stable stage of growth at approximate 78 h, and the maximum biomass of 49.1 g/L was obtained at 84 h. The biomass productivity for *Aurantiochytrium* sp. strain No. 32 was 0.58 g/L/h. Meanwhile, a maximum carbohydrate production of 31.3% DCW, reaching up to 15.4 g/L, was obtained at 84 h with carbohydrate productivity of up to 0.18 g/L/h. It was found that the carbohydrate content in the cell of *Aurantiochytrium* sp. strain No. 32 begun to be improved when the concentration of reducing sugar dropped to approximate 20 g/L and reached the highest level during the stable stage of the cell growth. This result suggested that the reducing sugar was first inflowed into the metabolic pathway for cell growth, and then into the carbohydrate synthesis for energy reservation when the reducing sugar was consumed to deficiency. Moreover, although microbial cell preferentially synthesizes lipid and carbohydrate under the nutrition conditions, lipid was accumulated more rapidly than carbohydrate. This finding could explain why the emergence of the maximum carbohydrate content in *Aurantiochytrium* sp. strain No. 32 (at 66 h) was later than that of the maximum lipid content in *Aurantiochytrium* sp. strain No. 6 (at 84 h), and carbohydrate fermentation needed more time for higher productivity [22,35]. This carbohydrate content in the *Aurantiochytrium* sp. strain No. 32 cell was comparable with or far higher than those of *Schizochytrium* sp. Strains [14,22,36]. *Aurantiochytrium* sp. has been widely applied as feed and nutrient-enriching composition in aquatic and livestock industries, due to the multiple nutrition in its cell, such as lipid-rich in PUFAs, carbohydrate, protein, and micronutrients [2,36]. Among these nutrients, carbohydrate is essential for animal cultivations, such as *Artemia franciscana*, oyster, scallop and cow, et al. [2,36,37]. Our result indicated that the *Aurantiochytrium* sp. strain No. 32, with a high growth rate and carbohydrate content, is fit for feed supplement in aquatic and livestock industries.

## 3. Materials and Methods

### 3.1. Materials

The nonadecanoicacid methyl ester used as the internal standard was purchased from Sigma-Aldrich (St. Louis, MO, USA). The BF_3_-methanol solution was purchased from ANPEL Laboratory Technologies Inc. (Shanghai, China). All other chemicals used in this study were of analytical grade and purchased from Aladdin Industrial Inc. (Shanghai, China).

### 3.2. Strains, Medium and Culture Conditions

A total of 109 *Aurantiochytrium* sp. strains from our *Aurantiochytrium* library, which was isolated from the mangrove ecosystem in Yueqing Bay (Zhejiang, China), were used in this study [26]. Each strain was preserved in 20% (*v*/*v*) glycerol and at −80 °C before it was used. The strain was transferred onto the GPY plate (2% glucose, 1% peptone, 0.5% yeast extract, 20% sea salt and 20% agar powder) for purification and recovering of strains. For screenings of strains with high levels of lipid, protein and carbohydrate contents, respectively, the single colony was transferred into the 250 mL flask containing 50 mL screening medium and cultured at 28 °C and 150 rpm for 3 days. The screening medium was composed of 8% glucose, 1% polypeptone, 0.5% yeast extract and 20% sea salt (*m*/*v*). To analyze the effect of deuteration distributed in cell on cellular contents of three biomacromolecules, the screening medium was prepared with H_2_O and 50% D_2_O, as the H_2_O medium and the D_2_O medium, respectively. The *Aurantiochytrium* sp. strain 6 was selected as the analyzed strain, and it was transferred into the D_2_O medium and cultured for deuteration of cell. The cultured cells under the D_2_O medium were washed and diluted with D_2_O and was set as the D_2_O-treated group (D group). Meanwhile, the cells cultured in the H_2_O medium and prepared with H_2_O were set as the H_2_O-treated group (H and the control group). The cells of two groups were analyzed by the FT-IR based on the method described below. The results from the two groups were compared and analyzed. 

### 3.3. FT-IR Spectroscopy Analysis

The FT-IR analysis was based on the method described by Giordano et al. with minor modifications [17]. The optical density (OD) of the cell under 600 nm for every culture sample was measured and calculated. A proper proportion of dilution with the blank medium was performed to make sure the OD_600_ of every sample as 0.1. The culture with OD_600_ value of 0.1 was harvested by centrifugation at 6000× *g* for 5 min. The supernatant was removed, and the pellet was then washed twice with sterile isotonic saline. After the final wash, a total of 50 µL isotonic saline was left, and the cell pellet was re-suspended in this volume. For FT-IR spectroscopy analysis, 30 µL of cell suspension for every sample was homogenized using pipettor in 96 well microplates and dried at 60 °C for 90 min. Spectra were acquired with a TENSOR 27 FT-IR spectrometer (Bruker Optics, Ettlingen, Germany) equipped with a High Throughput Screening eX-Tension (HTS-XT) unit. The spectral region was set 4000 to 600 cm^−1^ with a resolution of 4 cm^−1^, an aperture of 5.0 mm, taking 64 scans that were subsequently averaged. The spectra were resorted to baseline correction to minimize variations between spectra caused by the baseline shift. The OPUS 6.5 software (Bruker Optics, Billerica, MA, USA) package was used for quantitive and qualitative analysis of target peaks. 

Peak qualitative analysis was performed according to the method from Giordano et al. with minor modifications [17]. Absorption signals around 3000–2800 cm^−1^ and 1740 cm^−1^, representing the C–H stretching vibration acyl chains and the ester C=O stretching band, corresponding to the lipid content (Peaks 1, 2 and 3 in Figure 1). Absorption signals around 1690–1650 cm^−1^ and 1540–1500 cm^−1^ representing amide I and amide II, corresponds to protein C=O stretching vibration and N–H bending vibrations, respectively, were characterized as protein content (Peaks 4 and 5 in Figure 1). Absorption signals around 1200–900 cm^−1^ characterized over a C–O and C–O–C stretching band area, were used to quantify the total carbohydrate content in the cell (Peaks 6 and 7 in Figure 1). OD_600_ (0.1–0.3) of every sample was used for normalization of the results derived from the FT-IR.

### 3.4. Determination of Biomass

The biomass was determined as the dried cell weight (DCW) or the optical density under 600 nm (OD_600_). For DCW analysis, an aliquot of 50 mL of culture was centrifuged at 4 °C and 10,000× *g* for 5 min and then washed twice with distilled water. The cell pellet was lyophilized to a constant weight at 50 °C for 2 days. If needed, the dried cells were pestled into a fine powder with mortar under liquid nitrogen for further analysis. For OD_600_ analysis, proper dilution time of cell culture with a blank medium was performed to make sure the reading of spectrophotometer was at a range of 0.1–0.3. The OD_600_ of cell culture was then calculated.

### 3.5. Traditional Biochemical Analysis of Cellular Composition

Lipid, protein, and carbohydrate were analyzed using traditional biochemical methods to validate the FT-IR results. Lipid content analysis was performed according to our previous method [7]. The lyophilized cell was ground by mortar and pestle into a fine powder under liquid nitrogen. The cell powder was extracted into 100 mL of chloroform/methanol (2:1, *v*/*v*) at room temperature. The lipid extract was dried by evaporation and weighed. Carbohydrate content was analyzed using sulfuric acid-anthrone method [16]. Absorbance was measured at 620 nm with Ultrospec 2100 pro Spectrophotometer (GE, Boston, MA, USA). A calibration curve was prepared using glucose as a standard. Protein content was analyzed using the Bradford method [15]. Fatty acids analysis was performed based on our previous method [7]. 

### 3.6. Media Optimization for Lipid or Carbohydrate Productions

To optimize media composition for lipid and carbohydrate productions, glucose, peptone and yeast extract, representing carbon and nitrogen resources, were optimized through the “One at a time” method. Different concentrations of glucose (40, 60, 80, 100, 120 and 140 g/L), peptone (0.5, 1, 1.5, 2, 3 and 4 g/L) and yeast extract (0, 0.5, 1, 2, 3 and 4 g/L) were tested, and one factor was optimized when the other factors were fixed. The biomass, lipid, and carbohydrate, measured by the traditional method and FT-IR method, respectively, were responses for optimizations.

### 3.7. Fermentation in 5 L Bioreactor 

The RALF 5 l bioreactor (Bioengineering, Switzerland) was used to perform the fed-batch bioreactor cultivations using the optimal media obtained from above optimizations. The pre-culture was prepared in a 500 mL flask and then transferred into the 5 L bioreactor containing 4 l media optimized for lipid and carbohydrate production, respectively. For lipid production, the fermentation was set based on our previous method [26]. The temperature was set at 25 °C with aeration and agitation rate kept at 2 vvm and 600 rpm, respectively. For carbohydrate production, the fermentation temperature was set at 28 °C with aeration and agitation rate kept at 1 vvm and 400 rpm, respectively. Fifty milliliters of sample was taken at 6 h intervals over the entire cultivation (72 h for lipid fermentation and 96 h for carbohydrate fermentation) for DCW, reducing sugar, lipid, fatty acid, carbohydrate, and FT-IR analyses.

### 3.8. Statistical Analysis

All the experiments above were repeated three times, and the average values were taken as the results.

## 4. Conclusions

A bio-macromolecule analysis based on the FT-IR, specific for the oleaginous *Aurantiochytrium* sp., was firstly constructed in this study. This method was economic, green, and accurate, and was applied in strain screenings, medium optimization, and fermentation. Two *Aurantiochytrium* sp. strains were screened for lipid and carbohydrate productions, respectively. Lipid and carbohydrate accumulated rapidly when the concentration of the reducing sugar in medium consumed to deficiency, and accumulation of lipid was ahead of that of carbohydrate. The 5-l fermentation performance of the two strains confirmed their potentials for lipid, DHA, and carbohydrate production, and broad applications in medical, food, and feed industries.

## Figures and Tables

**Table 1:** Evaluation of the FT-IR method compared with the traditional biochemical methods for cellular composition analysis in *Aurantiochytrium* sp.

Evaluation Index	Traditional Biochemical Methods	FT-IR Method
Cultivation scale	50 mL (in 250 mL flash)	200 μL (in tube)
Sample condition	Lyophilized cells	Liquid sample
Time consumed	~72 h	~4 h
Chemical reagents	Liquid nitrogen, chloroform, and methanol for lipid; sulfuric acid and anthrone for carbohydrate	None
Equipment	Centrifuge, freeze dryer, mortar, pestle, evaporimeter, and balance	FT-IR spectrometer and spectrophotometer
Cost	Very high	Low
Accuracy	High	High
